# Molecular Simulations of Energy Materials

**DOI:** 10.3390/molecules30214270

**Published:** 2025-11-03

**Authors:** Viorel Chihaia, Godehard Sutmann

**Affiliations:** 1 Institute of Physical Chemistry “Ilie Murgulescu” of the Romanian Academy, Splaiul Independentei 202, Sector 6, 060021 Bucharest, Romania; 2 Jülich Supercomputing Centre (JSC), Institute for Advanced Simulation (IAS), Forschungszentrum Jülich, 52428 Jülich, Germany; 3 Interdisciplinary Centre for Advanced Materials Simulations (ICAMS), Ruhr Universität Bochum, 44801 Bochum, Germany

The accelerating demand for energy, coupled with the ongoing depletion of conventional energy resources and environmental problems, poses a critical challenge to the scientific community [1]. Addressing this challenge requires the development of innovative materials capable of generating, converting, storing, and utilizing energy in ways that are both sustainable and environmentally benign [2]. Understanding these complex systems—spanning diverse phenomena and interacting across multiple spatial (from atomic to macroscopic) and temporal (from femtoseconds to years) scales—demands an integrated scientific approach [3]. While experimental research remains essential in uncovering the behavior of energy materials, especially under harsh environmental conditions, many microscopic-scale mechanisms remain poorly understood [4]. This is where molecular-level computational simulations can play an important role. Advances in computer molecular sciences now offer powerful methods for probing the structure, dynamics, and reactivity of materials at the atomic and molecular levels, complementing experimental findings and offering predictive insights [5]. In particular, molecular simulations—encompassing static modeling, molecular dynamics, and Monte Carlo methods—enable the exploration of energy materials under various conditions [6]. These approaches can operate across quantum, classical, and coarse-grained frameworks, each providing valuable perspectives on intra- and intermolecular forces. Quantum mechanical methods reveal critical details of electronic structure, which underpin macroscopic properties and device performance [7], while atomistic and coarse-grained simulations offer scalable insights into larger systems and longer-time-scale processes [8]. To fully capture the multiscale nature of energy materials, there is a growing need to integrate particle-based methods with continuum models through multiresolution and multiscale approaches [9,10]. Such hybrid strategies promise to deepen our understanding of the fundamental phenomena governing the behavior of materials in real-world energy and environmental applications.

This Special Issue aims to highlight recent advances in atomic-scale simulation methods and their application to energy materials science. Contributions demonstrate how computational tools provide crucial insights into the design, characterization, and optimization of materials for a sustainable energy future. The main investigated properties and phenomena are summarized in Table 1.

Hydrogen production through solar-driven photoelectrochemical (PEC) water splitting represents one of the most promising pathways toward sustainable energy. However, traditional semiconductors such as titanium dioxide (TiO_2_) face key limitations, such as a wide bandgap and poor conductivity. In this context, Akbar et al. (contribution 1) employ density functional theory (DFT) to explore how doping TiO_2_ with transition metals (Ag, Fe, and Co) can improve its visible-light absorption and charge transport. Their comparative analysis of dopants, using GGA+U and hybrid functionals, illustrates how atomistic modifications influence electronic structure, optical response, and mechanical stability—ultimately guiding the rational design of efficient photocatalysts.

Alongside efforts in hydrogen production, the search for cost-effective electrocatalysts for fuel cells remains a critical area of research. Fe–N–C systems, especially FeN_4_-doped graphene nanoribbons (GNRs), have emerged as high-potential non-precious metal catalysts for oxygen reduction reactions (ORRs). Mineva et al. (contribution 2) present a DFT-based study on how dopant position, edge termination, and spin ordering affect the electronic and magnetic properties of FeN_4_-GNR systems. Their work provides insights into spin polarization control and its implications for catalytic activity, highlighting potential applications in spintronic devices and magneto-electronic catalysis.

Hydrogen separation technologies are equally crucial in advancing the hydrogen economy. Rosen and Sohlberg (contribution 3) investigate dual-phase perovskite materials (BaCe_0.85_Fe_0.15_O_3−δ_/BaCe_0.15_Fe_0.85_O_3−δ_) using DFT and first-principles thermodynamics. Their work focuses on surface reduction, vacancy formation, and thermodynamic stability under hydrogen-rich conditions. By constructing Gibbs free energy profiles and analyzing defect behavior, their study informs the design of stable, high-performance materials for applications such as proton-conducting membranes and hydrogen purification systems.

The importance of solvation effects in photophysics is explored by Manian et al. (contribution 4), who focus on xanthione—a sulfur-containing polycyclic aromatic compound known for its unusual anti-Kasha behavior and high solvent sensitivity. Through a hybrid approach combining quantum chemistry and MD simulations, the authors assess how different solvation models (implicit vs. explicit) impact the molecule’s excited-state dynamics. Their findings emphasize the necessity of explicit solvent modeling for accurately capturing the photophysical behavior of solvent-sensitive materials, with implications for quantum photonics, molecular electronics, and light-harvesting systems.

The intersection of quantum mechanics and energy storage is exemplified by Liu and Hanna (contribution 5), who explore quantum batteries—devices that utilize quantum coherence to store and transfer energy via excitonic mechanisms. Their simulation-based study introduces a symmetry-enabled open quantum network model, revealing how exchange symmetry and decoherence affect energy retention and transfer dynamics. This work opens new directions in quantum energy material research, bridging condensed matter physics and nanoscale energy design.

On the molecular scale, wettability and interfacial dynamics play a critical role in enhanced oil recovery (EOR). Ahmadi and Chen (contribution 6) use MD simulations to investigate how asphaltene concentration in oil droplets affects their adsorption on quartz surfaces, particularly under high-temperature conditions relevant to Steam-Assisted Gravity Drainage (SAGD). Their study uncovers the interplay between oil composition, temperature, and surface interactions, offering mechanistic insights into thermally induced wettability alteration and interfacial behavior in heavy oil reservoirs.

Subsurface challenges such as solid production in carbonate reservoirs can be addressed through atomistic modeling. Hue et al. (contribution 7) simulate the adsorption of hydrolyzed polyacrylamide (HPAM) on calcite surfaces using classical MD in isothermal–isobaric ensembles. Their results provide critical data for designing polymer-based additives to stabilize formation rock and prevent damage, optimizing EOR strategies under varying geochemical conditions.

Grenev and colleagues (contribution 8) conducted a computational screening study to identify metal–organic frameworks (MOFs) and zeolites with high potential for helium/nitrogen (He/N_2_) separation—an increasingly important challenge in industrial gas purification. Given helium’s vital role in fields such as cryogenics and nuclear technology, along with its growing scarcity, there is an urgent need for efficient, selective, and cost-effective separation materials. This study highlights the power of high-throughput molecular simulations in accelerating the discovery of porous materials with optimized pore architectures and sorption properties tailored to specific gas separation tasks. By screening over 10,000 MOFs and 218 zeolites, Grenev and colleagues demonstrated the ability to identify materials with superior adsorption and membrane selectivity for helium/nitrogen separation. The computational approach not only pinpointed the top-performing frameworks but also established structure–performance relationships, such as optimal pore-limiting diameters and accessible surface areas, which are critical for efficient separation. This methodology exemplifies how computational tools can streamline the design and selection of materials for industrial applications, reducing reliance on costly and time-consuming experimental trials.

This Special Issue highlights how first-principles and molecular dynamics simulations are transforming energy research across scales—from electronic structure and spin dynamics to interfacial phenomena and bulk material performance. Each contribution demonstrates the value of atomistic insights in guiding experimental design, improving material functionality, and enabling innovation in sustainable energy technologies. As computational power and theoretical models continue to evolve, molecular simulation will remain a cornerstone of next-generation material science and molecular engineering.

## Figures and Tables

**Table 1:** The properties, phenomena, and simulation methods involved in the articles of the present Special Issue dedicated to molecular simulations for energy materials.

Contribution	Topic	Properties Studied	Phenomena	Simulation Methods ^a^
1	Doped TiO_2_ (Ag, Fe, Co) for water splitting	Band gap, elastic constants, mechanical stability	Doping, water dissociation, light absorption, photoconductivity	DFT calculations
2	FeN_4_-doped graphene nanoribbons	Spin distribution, electronic structure, magnetism	Effect of dopant position and edge termination on electronic/magnetic properties	Spin-polarized DFT
3	Mixed-metal oxide perovskites (BaCe–Fe–O)	Thermodynamic stability, surface reduction energy, oxygen vacancy behavior	Surface dehydration, H_2_ interaction, defect formation	DFT-based thermodynamics
4	Xanthione in polar solvents	Electronic transitions, solvent sensitivity, photostability	Solvent effect on excited states, non-Kasha behavior	TDDFT, QC-MD, explicit and implicit solvent
5	Quantum battery network	Exciton population, energy retention, site energy influence	Dark state storage, discharge dynamics, exciton transfer	Open quantum network theory, quantum dynamics simulations
6	SAGD process–bitumen/quartz interaction	Wettability, adsorption energy, surface affinity	Wetting alteration under high temperature, asphaltene adsorption	CFF-MD, with varying conditions
7	HPAM polymer on calcite	Adsorption behavior, interaction strength, effect of ionic environment	Polymer adsorption, salt-bridging, charge screening	CFF-MD simulations
8	MOFs and zeolites for He/N_2_ separation	Henry’s constant, diffusion coefficients, selectivity, permeability	Gas adsorption, diffusion, and membrane-based separation	GCMC, Equilibrium CFF-MD

^a^ see the short notations listed in the Abbreviations section.

## Abbreviations

The following abbreviations are used in this manuscript:

DFT	Density Functional Theory
TDDFT	Time-Dependent DFT
QC	Quantum Chemical
CFF	Classical Force-Field
GO	Geometry Optimization
MC	Monte Carlo
GCMC	Grand Canonical Monte Carlo
MD	Molecular Dynamics
SAGD	Steam-Assisted Gravity Drainage
HPAM	Hydrolyzed Polyacrylamide
MOF	Metal–Organic Frameworks
